# TGF-β dependent regulation of oxygen radicals during transdifferentiation of activated hepatic stellate cells to myofibroblastoid cells

**DOI:** 10.1186/1476-5926-6-1

**Published:** 2007-02-20

**Authors:** Verena Proell, Irene Carmona-Cuenca, Miguel M Murillo, Heidemarie Huber, Isabel Fabregat, Wolfgang Mikulits

**Affiliations:** 1 Department of Medicine I, Division: Institute of Cancer Research, Medical University of Vienna, Borschke-Gasse 8a, A-1090 Vienna, Austria; 2 Departamento de Bioquímica y Biología Molecular, Facultad de Farmacia, Universidad Complutense de Madrid, Madrid 28040, Spain; 3 IDIBELL-Institut de Recerca Oncològica, Gran Via s/n, Km 2.7, L'Hospitalet, Barcelona, Spain

## Abstract

**Background:**

The activation of hepatic stellate cells (HSCs) plays a pivotal role during liver injury because the resulting myofibroblasts (MFBs) are mainly responsible for connective tissue re-assembly. MFBs represent therefore cellular targets for anti-fibrotic therapy. In this study, we employed activated HSCs, termed M1-4HSCs, whose transdifferentiation to myofibroblastoid cells (named M-HTs) depends on transforming growth factor (TGF)-β. We analyzed the oxidative stress induced by TGF-β and examined cellular defense mechanisms upon transdifferentiation of HSCs to M-HTs.

**Results:**

We found reactive oxygen species (ROS) significantly upregulated in M1-4HSCs within 72 hours of TGF-β administration. In contrast, M-HTs harbored lower intracellular ROS content than M1-4HSCs, despite of elevated NADPH oxidase activity. These observations indicated an upregulation of cellular defense mechanisms in order to protect cells from harmful consequences caused by oxidative stress. In line with this hypothesis, superoxide dismutase activation provided the resistance to augmented radical production in M-HTs, and glutathione rather than catalase was responsible for intracellular hydrogen peroxide removal. Finally, the TGF-β/NADPH oxidase mediated ROS production correlated with the upregulation of AP-1 as well as platelet-derived growth factor receptor subunits, which points to important contributions in establishing antioxidant defense.

**Conclusion:**

The data provide evidence that TGF-β induces NADPH oxidase activity which causes radical production upon the transdifferentiation of activated HSCs to M-HTs. Myofibroblastoid cells are equipped with high levels of superoxide dismutase activity as well as glutathione to counterbalance NADPH oxidase dependent oxidative stress and to avoid cellular damage.

## Background

Antioxidant defense mechanisms evolved as a consequence of the aerobic lifestyle caused by the photosynthetic activity of herbal organisms, which in turn depends on the capability of oxygen reduction occurring during respiration. Reactive oxygen species (ROS) are essential for a couple of processes within the cell and play a critical role in several diseases including liver damage [1]. ROS are produced (i) by the interaction of ionizing radiation with biological molecules, (ii) during cellular respiration and (iii) by myeloperoxidase and nicotinamide-adenine dinucleotide phosphate (NADPH) oxidase of phagocytic cells such as neutrophils and macrophages. In addition, several non-phagocytotic cell types such as hepatocytes [2] and hepatic stellate cells (HSCs) [3] have also been shown to express a NADPH oxidase-like enzyme playing an important role in the generation of ROS [4].

Strong oxidants like ROS can damage proteins, lipids (lipidperoxidation) as well as DNA, and therefore have been suggested to have a critical implication in carcinogenesis [5]. As a consequence, each cell type harbors several defense mechanisms against the noxious effects of oxidative stress. Two enzymes play a major protective role, namely the superoxide dismutase (SOD), which converts two superoxide anions (O_2_^-^) into hydrogen peroxide (H_2_O_2_) and oxygen, and catalase, which promotes the conversion of hydrogen peroxide to water and molecular oxygen. Antioxidants such as ascorbic acid, β-carotene and α-tocopherol also reduce danger from accidentally produced ROS. Another defense mechanism is based on glutathione (γ-glutamyl-cysteinyl-glycine, GSH), which participates in many different cellular actions including nutrient metabolism and regulation of cellular events such as signal transduction, cytokine production, cell proliferation, apoptosis and immune response [6]. However, GSH is mainly known as an intracellular redox system exhibiting two conformations, the antioxidant "reduced glutathione" tripeptide conventionally termed as the above mentioned GSH, and the oxidized form, a sulfur-sulfur linked compound known as glutathione disulfide (GSSG).

Apart from putative harmful consequences caused by ROS, recent reports demonstrate that free radicals are also implicated in cell signaling, especially in tumor cells and cells determined to undergo apoptosis. There exist strong evidence particularly for liver diseases that increased production of free radicals and/or impaired antioxidant defense mechanisms are involved. As a consequence, numerous studies have been focused on the pathological significance of ROS in liver injury as well as on therapeutic intervention with antioxidants [1,7-10].

Hepatic stellate cells play a pivotal role during liver injury. In the adult healthy liver, HSCs are considered as the principal storage site of retinoids, whereas HSCs get activated to myofibroblasts (MFBs) upon liver damage. This transdifferentiation is accompanied by drastic morphological changes including loss of cytoplasmic lipid droplets and alterations in protein synthesis patterns, which comprises *de novo *synthesis of α-smooth muscle actin [11-14]. Furthermore, HSC-derived MFBs are mainly responsible for extracellular matrix (ECM) remodeling in the fibrotic liver, which represents a hallmark of fibrogenesis. In particular, MFBs secrete high levels of the interstitial collagens I and III [15] as well as several matrix metalloproteinases (MMPs) [14,16] and tissue inhibitors of MMPs [16-18], resulting in a dense and rigid network of matrix constituents which exerts physical stress on surrounding cells.

Whether ROS are implicated in HSC activation and which molecular mechanisms are the basis for the transdifferentiation of HSCs to MFBs is still a matter of debate. Lee and colleagues demonstrated that ROS are indispensable for HSCs activation and that c-myc and NF-κB act as molecular mediators of oxidative stress [19]. In addition, co-culture experiments have shown that extracellular ROS, produced by stable cytochrome P450 2E1 (CYP2E1) overexpression in HepG2 cells, facilitate activation of quiescent HSCs *in vitro*, resulting in increased expression of collagen I and α-SMA [20]. Moreover, treatment of hepatocytes with nitrilotriacetate complex results in oxidative stress response. It has been shown that transfer of conditioned medium on HSCs stimulated their proliferation as well as collagen I accumulation within these cells [21]. Similar results were obtained in Kupffer and other inflammatory cells, which have been shown to produce H_2_O_2 _[19,22,23].

One of the most extensively studied antagonistic player of ROS is GSH, which has been reported to be significantly upregulated in cultured primary HSCs at day seven compared to freshly isolated HSCs [24]. In addition, long-term cultured HSCs exhibit a higher synthesis rate of GSH compared to cells in short-term culture. In contrast, no increased GSH or γ-glutamyl-cysteine synthetase (GCS) level has been observed in isolated HSCs from fibrotic rat livers after 8 weeks of bile duct ligation or 4 weeks of CCl_4 _treatment [24].

Recently, we published a hepatic stellate cell line referred to as M1-4HSC [25], which has been isolated from p19^ARF ^null mice and represents activated HSCs displaying an amazing plasticity concerning their morphology. Since they have undergone spontaneous activation *in vitro*, M1-4HSCs have already lost fat-storing droplets and express high amounts of α-SMA. Due to TGF-β administration, these cells are provoked to undergo a further activation process to myofibroblastoid cells, termed M-HTs [25,26]. Hence, this cellular model provides the unique ability to study late stage events of HSCs activation, i.e. the transdifferentiation of activated HSCs to MFBs. Indeed, most studies investigating HSC activation have employed freshly isolated, quiescent HSCs and monitored spontaneous activation which takes place as soon as cells are cultured *in vitro*, whereby TGF-β is suggested to accelerate transdifferentiation even though it is not required [27].

We addressed the question whether oxidative stress is implicated in late stage activation of M1-4HSCs to myofibroblastoid M-HTs. In order to elucidate whether ROS plays a role in TGF-β driven transdifferentiation, we monitored ROS levels during the first 72 hours of TGF-β treatment, which is referred to as induction phase. We show that ROS are upregulated during TGF-β driven HSCs activation, whereas M-HTs displayed a very effective counterregulation to TGF-β induced oxidative stress by upregulation of SOD enzymatic activity rather than catalase. In addition, genes implicated in the response to oxidative stress such as c-fos and c-jun as well as platelet-derived growth factor (PDGF) receptors α and β are shown to be regulated which points to their regulatory functions in establishing resistance to oxidative stress.

## Results and discussion

### Increase of ROS levels during the induction phase of TGF-β driven M1-4HSCs activation to MFBs

**Figure 1:**
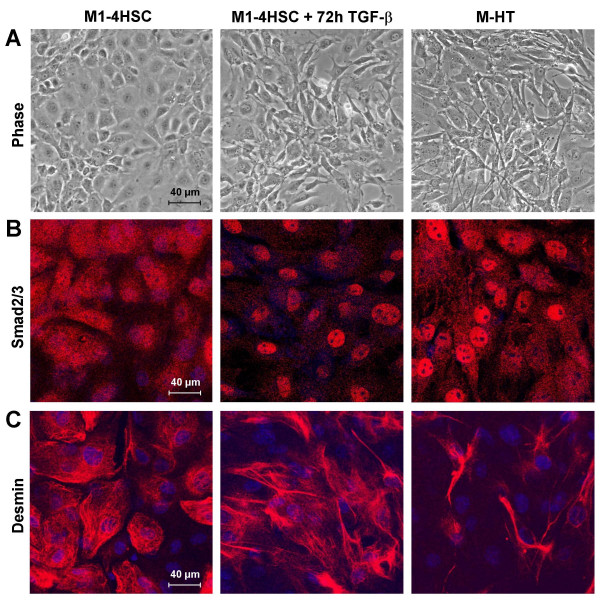
**Cellular model of hepatic fibrosis**. (A) Morphological changes of M1-4HSCs treated with TGF-β1 either for 72 hours or for long-term (myofibroblastoid M-HT) as analyzed by phase contrast microscopy. (B) Nuclear translocation of Smad2/3 as visualized by confocal immunofluorescence analysis. (C) Confocal immunofluorescence images after staining of cells with anti-desmin antibody.

**Figure 2:**
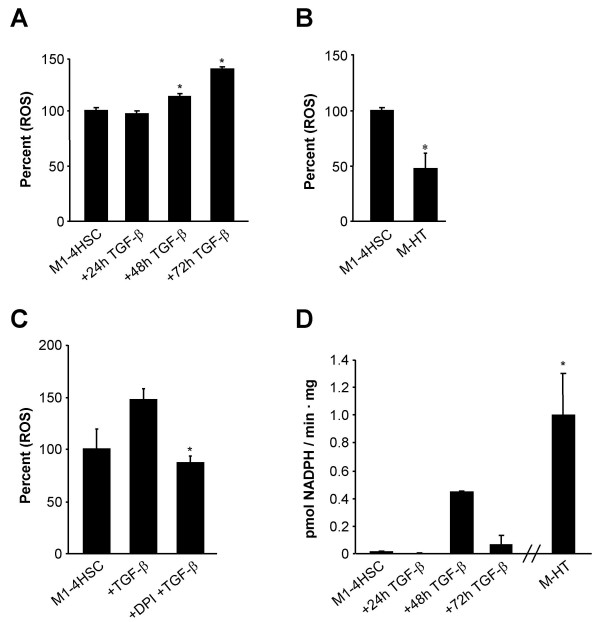
**TGF-β mediated accumulation of ROS associates with increased NADPH oxidase activity**. (A) During the TGF-β dependent transdifferentiation of M1-4HSCs, ROS levels increase after 48 and 72 hours. (B) M-HTs show a reduction of ROS levels to about 50% as compared to untreated M1-4HSCs. (C) DPI inhibits TGF-β caused ROS accumulation in M1-4HSCs. (D) TGF-β treatment of M1-4HSCs induces NADPH oxidase activity after 48 hours. M-HTs display vast NADPH oxidase activity. For all situations, n = 3. * p < 0.05.

The cell line M1-4HSC represents activated HSCs, which undergo further activation to myofibroblast-like cells in response to TGF-β [25]. The induction phase refers to 72 hours of TGF-β treatment, which is characterized by the change to a myofibroblastoid morphology (Fig. 1A). After 20 days of TGF-β administration, the cells represent activated MFBs with a stable phenotype, termed M-HTs. Transdifferentiation of M1-4HSCs to M-HTs shows increased nuclear accumulation of Smad2/3 (Fig. 1B), indicating a further activation of TGF-β signaling. In addition, M-HTs exhibit decreased expression of desmin (Fig. 1C), as reported recently [25]. This cellular model provides the unique ability to monitor late stage events during fibrogenesis, since spontaneous activation has already occurred. In order to examine whether ROS are implicated in this transdifferentiation from activated HSCs to MFBs, we analyzed intracellular hydrogen peroxide during the induction phase compared to untreated M1-4HSCs and M-HTs. Hydrogen peroxide was used as a general marker of oxidative stress since all forms of oxygen radicals that occur intracellularly are finally converted into H_2_O_2_. We observed a significant increase in ROS levels after 48 and 72 hours of TGF-β treatment (Fig. 2A), whereas no elevation of hydrogen peroxide levels was determined after 24 hours. Since basal levels of ROS are already induced in M1-4HSCs compared to quiescent HSCs, as reported by several investigators [20,28-30], TGF-β is obviously able to provide accumulation of hydrogen peroxide in M1-4HSCs. In contrast, M-HTs showed about 40% reduced intracellular hydrogen peroxide content compared to untreated M1-4HSCs (Fig. 2B). Hence, these data raised the question whether the lowered ROS levels in M-HTs were caused by reduced ROS production or by the upregulation of cellular antioxidant defense mechanisms. To properly tackle this issue we asked for the major source of ROS in M1-4HSCs caused by TGF-β administration.

### TGF-β treatment of M1-4HSCs results in induction of NADPH oxidase activity

In most cell types, mitochondria-anchored enzymes provide the majority of ROS such as NADPH-ubiquinone oxidoreductase and ubiquinol cytochrome oxidoreductase [31]. Another important source for ROS is NADPH oxidase, which has been shown to be active in several non-phagocytotic cell types including HSCs [32]. This NADPH oxidase-like enzyme is a multi-protein complex consisting of the transmembrane proteins p22^phox ^and the p91^phox^-related enzymes of the NADPH oxidase (Nox) family, the cytosolic proteins p47^phox ^and p67^phox ^as well as the small GTP binding protein Rac. NADPH oxidase activity depends amongst others on the co-enzyme flavin and can be therefore inhibited by diphenyleneiodonium chloride (DPI). The involvement of NADPH oxidase in the TGF-β-dependent increase of oxidative stress in M1-4HSC was obtained by measurements of ROS content in cells that have been treated with TGF-β 1 in the presence of DPI. M1-4HSC starved for 6 hours and administrated with TGF-β 1 for 3 hours resulted in an increase of ROS levels to 50% (Fig. 2C). This accumulation of oxidative stress was impaired by simultaneous co-incubation with TGF-β and DPI, as ROS levels under these conditions were comparable to those measured in control cells.

**Figure 3:**
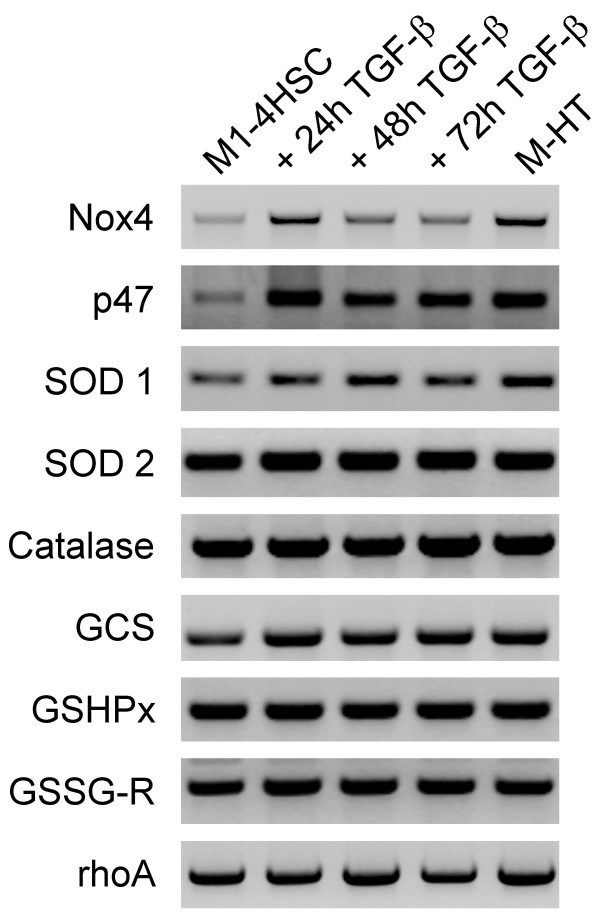
**Expression profiling of oxidative stress components by semiquantitative RT-PCR**. GCS, γ-glutamylcysteine synthetase; GSHPx, glutathione peroxidase; GSSG-R, glutathione reductase; SOD 1, Cu/Zn superoxide dismutase; SOD 2, mitochondrial superoxide dismutase. The constitutive expression of rhoA is shown as loading control.

Hence, we analyzed whether NADPH oxidase activity was affected in response to TGF-β1. The analysis revealed a strong elevation of NADPH oxidase activity after 48 hours which decreased again after 72 hours (Fig. 2D). In agreement with ROS levels observed at 24 hours, no NADPH oxidase activity could be detected. In contrast, myofibroblastoid M-HTs exhibited a highly elevated activity of the ROS producing enzyme compared to untreated M1-4HSCs. These results excluded that the intracellular hydrogen peroxide levels in these cells were caused by a low production of free radicals. In line with these data, the components of NADPH oxidase were found to be differentially transcribed. Control M1-4HSC and those treated with TGF-β as well as M-HTs were analyzed for NADPH oxidase components via linear, semi-quantitative RT-PCR. RhoA was used as control for RT-PCR because no variations in expression levels have been found upon transdifferentiation of M1-4HSCs, as described recently [25]. Both Nox4 and p47^phox ^were significantly upregulated during the induction phase of M-1HSCs activation to M-HTs (Fig. 3). Nox4 and p47^phox ^mRNA levels were already augmented after 24 hours of TGF-β1 administration although NADPH oxidase activity was increased 48 hours post TGF-β1 treatment. This discrepancy might be explained by the fact that transcription precedes translation and functional activation of the enzyme. Nox4 transcript levels were also found to be elevated in M-HTs (Fig. 3), which was in line with the high NADPH oxidase activity (Fig. 2D). Notably, Nox1, gp91^phox ^(Nox2) and Nox3 showed comparably enhanced mRNA levels in M-HTs (data not shown).

Taken together, these data point to a direct influence of TGF-β on NADPH oxidase activity and subsequent ROS accumulation during the transdifferentiation of M1-4HSCs to MFBs. According to the literature, upregulation of ROS due to TGF-β has also been shown in various cell types such as vascular smooth muscle cells [33], hepatocytes [34], fetal lung fibroblasts [35], cardiac fibroblasts [36] and also HSCs [28], most frequently by upregulation of NADPH oxidase activity [33,35-37]. However, the data available for HSCs refer to (i) the activation of quiescent HSCs and (ii) to proportionally short incubation times in comparison to the fibrosis model employed in this study. We focused on the induction phase within 72 hours compared to untreated, but already spontaneously activated parental M1-4HSCs and M-HTs, the latter grown for long-term in TGF-β supplemented medium and comparable to HSC-derived MFBs *in vivo*. These cells display very high NADPH oxidase activity as well as increased p47^phox ^and Nox mRNA despite of diminished levels of free radicals. Accordingly, Bataller *et al*. have recently shown the transcriptional upregulation of p47^phox ^in quiescent HSCs and activated HSCs isolated from healthy and cirrhotic rat livers, respectively [32]. In order to clarify the contradiction of reduced oxidative stress in M-HTs with a concomitant high activity of NADPH oxidase, we addressed the question for the regulation of counteracting mechanisms.

### Enzymatic defense mechanisms reduce TGF-β induced oxidative stress in M-HTs

**Figure 4:**
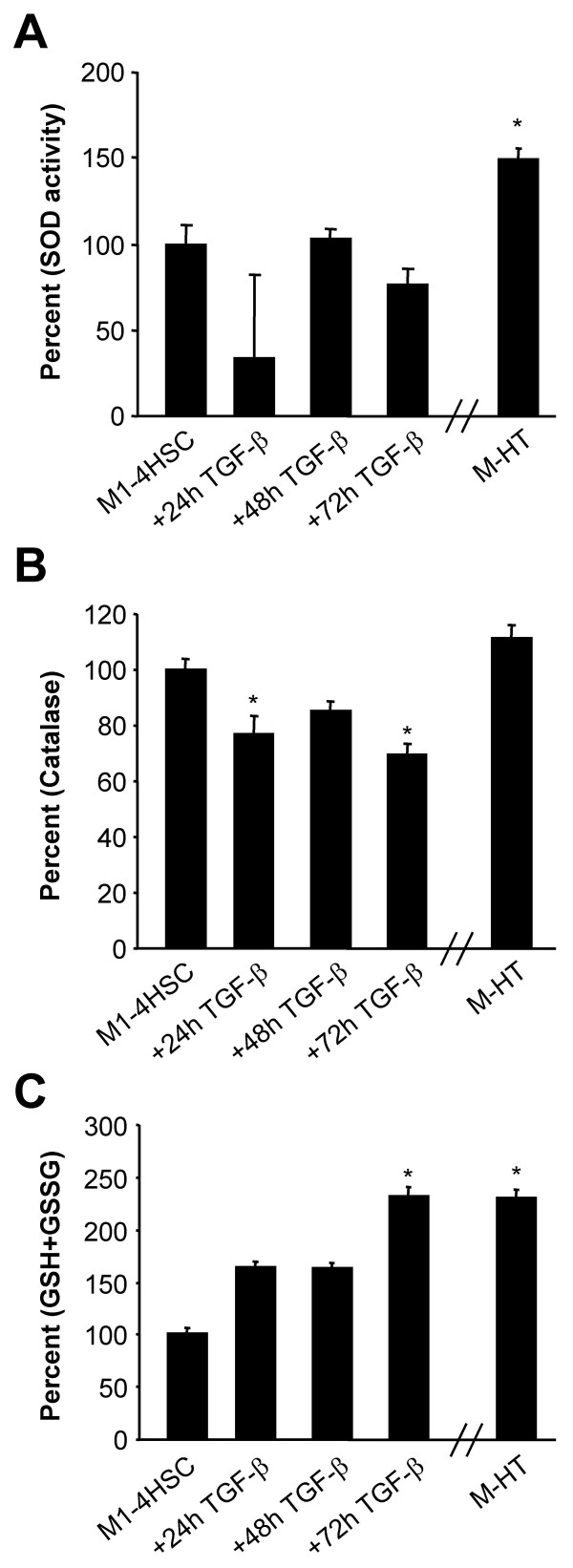
**Regulation of defense mechanisms against oxidative stress during the TGF-β driven transdifferentiation of M1-4HSCs to M-HTs**. (A) SOD activity (n = 2). (B) Catalase activity (n = 4). (C) Glutathione levels (n = 3). * p < 0.05.

To examine whether enzymatic defense strategies participate in the protection against intracellular ROS accumulation, we analyzed alterations in the enzyme activity of SOD. The superoxide anion O_2_^- ^is produced by NADPH oxidase and arises as free radical through leaking away from respiratory chain. In mammals, three SOD isoforms have been identified such as cytosolic Cu/Zn-SOD (SOD 1), mitochondrial Mn-SOD (SOD 2), and extracellular Cu/Zn SOD (SOD 3), which are responsible for the destruction of O_2_^- ^to hydrogen peroxide and oxygen. Subsequently, catalase and/or GSH redox cycle are responsible for removing hydrogen peroxide from the cell with water and oxygen as products. No significant alteration in SOD activity was observed during initiation of TGF-β driven MFB activation, whereas SOD activity in M-HTs was upregulated to 50% as compared to M1-4HSC (Fig. 4A). RT-PCR revealed a slight upregulation of SOD 1 mRNA during induction phase and showed highest expression in M-HTs (Fig. 3). The expression of SOD 2 mRNA also slightly increased after TGF-β treatment of M1-4HSCs. The high level expression of SOD 2 transcripts maintained upon kinetics of TGF-β administration and was even observed in M-HTs. An increase in SOD1 expression might produce a gain in the cytosolic SOD activity, which counteracts ROS production at the plasma membrane level. These results are in line with data obtained by the analysis of NADPH oxidase, which showed strongly enhanced activity in M-HTs, indicating huge amounts of superoxide anion that has to be removed mainly by the involvement of SOD.

Taken together these results point to an essential role for SOD 1 in M-HTs, facing an augmented superoxide anion content that has to be removed in order to protect cells from unfavorable consequences. This is essential even though oxidative stress supports the establishment of HSC activation and fibrosis, which has also been shown for stellate cells in the pancreas (PSC). For instance, Emori et al. reported an important role of SOD in PSC activation, as blocking by diethyldithiocarbamate resulted in a significant induction of α-SMA positive cells [38]. Therefore, we consider that TGF-β induced elevation of ROS is crucial for the transdifferentiation of M1-4HSCs to M-HTs. However, MFBs also depend on the reduction of free radical accumulation in order to survive.

### Catalase fails to resist elevated hydrogen peroxide levels

In order to reduce oxidative stress, intracellular H_2_O_2 _is dismutated to water and oxygen either by catalase or GSH redox cycle. Interestingly, we observed a slight downregulation of catalase activity during induction phase and a moderate upregulation in M-HTs as compared to parental M1-4HSCs (Fig. 4B). Corresponding mRNA levels were not regulated at all (Fig. 3) which leads to the conclusion that catalase is not crucially involved in oxidative stress defense during M1-4HSCs activation to M-HTs. These data are contrary to Bleser *et al*. who demonstrated that catalase mRNA levels where strongly induced in activated HSCs *in vivo *as well as *in vitro*. Therefore, we suggest that catalase induction represents an early event in HSCs activation, which does not participate in the counterregulation of oxidative stress in M-HTs. Previous data indicate a discriminating role between low and high concentration of H_2_O_2_, determining whether catalase or GSH redox cycle is more likely to clear free radicals. In general, it is proposed that GSH is more efficient at low intracellular H_2_O_2 _concentrations whereas high amounts of H_2_O_2 _are preferentially removed by catalase [29,39-41]. This points to rather moderate H_2_O_2 _levels in M1-4HSCs, which might be important in signal transduction supporting the late stage activation to M-HTs. Therefore, we hypothesized that the GSH redox cycle must have considerable implications in developing resistance to ROS in M-HTs.

### Glutathione upregulation refers resistance to TGF-β induced oxidative stress in activated HSCs

Among various other functions, GSH is mainly involved in the maintenance of the intracellular redox homeostasis including removal of hydrogen peroxide. We found that total glutathione levels were upregulated after 24 and 48 hours, and were even more than doubled after 72 hours of TGF-β treatment (Fig. 4C). This elevation in intracellular glutathione content was further detected in M-HTs, which exhibited a 2.5 fold higher level than untreated M1-4HSCs. Moreover, we analyzed whether the expression of redox cycle components are affected. Noteworthy, the production of glutathione is achieved by *de novo *synthesis through synthetases such as γ-glutamyl-cysteine synthetase (GCS). Interestingly, RT-PCR analyses of the corresponding transcript showed that GCS was slightly induced after 24 hours TGF-β treatment and maintained elevated in M-HTs (Fig. 3). In addition, we examined the mRNA level of glutathione peroxidase (GSHPx) and glutathione reductase (GSSG-R), which are suggested to be involved in removing peroxides (using GSH as substrate) and reducing GSSG, respectively. However, no modulation of transcript levels was found.

Taken together, these results suggest a direct regulation of NADPH oxidase by TGF-β and increased ROS levels as well as a particular contribution of GSH in the resistance to augmented oxidative stress. Interestingly, Bleser *et al*. proposed catalase to be more effective to remove high local concentrations of ROS, which are represented by intracellular produced H_2_O_2_. Contrary, extracellular H_2_O_2_results in consumption of GSH. This might be true for the early activation phase of quiescent HSCs but not for the completion of transdifferentiation to MFBs. Since catalase activity was slightly downregulated during 72 hours of TGF-β treatment and reached a moderate activity in M-HTs, catalase might not be effective in removing H_2_O_2_. In conclusion, these data indicate that SOD activity is responsible for reduction of oxidative stress in M-HTs in cooperation with GSH. In order to gain insight into how these pathways might be regulated, we analyzed target genes that are involved in the response to oxidative stress.

### Transcriptional upregulation of AP-1 transcription factors and PDGF receptor subunits during HSCs activation to myofibroblastoid M-HTs

**Figure 5:**
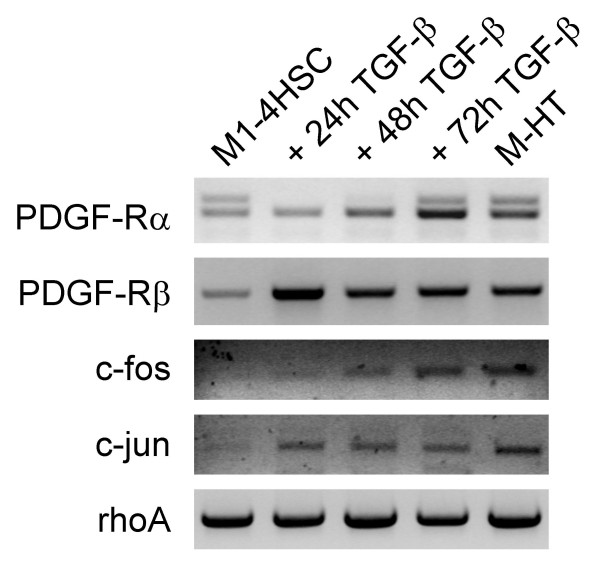
**Steady state transcript levels of PDGF receptors and AP-1 components as analyzed by semiquantitative RT-PCR**. The constitutive expression of rhoA is shown as loading control.

Since AP-1 transcription factor is involved in stress response, we examined the regulation of its subunits c-fos and c-jun by RT-PCR during the transdifferentiation of M1-4HSCs to M-HTs. The upregulation of both mRNAs was maintained in M-HTs (Fig. 5), which points to a regulatory function of AP-1 involved in establishing resistance to oxidative stress. Since it has been shown that PDGF is regulated upon oxidative stress [3], we determined mRNA levels of PDGF receptors α and β in M1-4HSCs. Indeed, PDGF receptor transcripts were increased within the induction phase, since upregulation of PDGF-Rα mRNA was detected after 48 hours and even further increased after 72 hours as well as in M-HTs. Unlike PDGF-Rα, whose mRNA levels were not affected after 24 hours TGF-β administration, PDGF-Rβ mRNA abundance already peaked at 24 hours with a more than 10-fold induction. Besides its well known function as potent mitogen, PDGF is implicated in numerous other processes including wound healing and the formation of connective tissue by stimulating the production of several matrix molecules such as collagens and fibronectin [42]. This is in accordance with our data since HSC-derived M-HTs secrete vast amounts of these ECM components (unpublished data), which mimics the *in vivo *situation during liver fibrogenesis. In addition, it has been shown by Adachi *et al*. that PDGF-BB ligand induces NADPH oxidase to produce ROS, which in turn stimulates proliferation of LI-90 cells [3]. Thus, the upregulation of PDGF-Rβ expression might contribute to the increase of NADPH oxidase activity in M-HTs.

In summary, we show that even though M-HTs harbor hyperactive NADPH oxidase, these myofibroblastoid derivatives of M1-4HSCs have reduced ROS levels compared to the untreated cell line. The cellular antioxidant defense mechanism depends on the increased activity of SOD, which converts the free radical O_2_^- ^to hydrogen peroxide that is subsequently reduced either by the GSH redox cycle or by catalase. Since catalase does not seem to be affected during this process of HSC activation, we suggest that the resistance to oxidative stress in M-HTs hinges on the significantly increased availability of GSH.

## Conclusion

The current investigation demonstrates the TGF-β dependent production of reactive oxygen species upon transdifferentiation of derivatives of hepatic stellate cells (M1-4HSC line) to M-HTs. The data provide evidence that (i) the increase of oxidative stress correlates with a gain in NADPH oxidase activity, and (ii) superoxide dismutase activation in cooperation with glutathione reduces radical accumulation in myofibroblastoid cells. These defense mechanisms are suggested to be particularly relevant in order to protect myofibroblastoid cells from harmful consequences caused by oxidative stress.

## Materials and methods

### Cell lines

M1-4HSC and derivative M-HT lines were grown in DMEM plus 10% fetal calf serum (FCS) as described previously [25]. M-HTs were additionally supplemented with 1 ng/ml TGF-β1 (R&D Systems, Minneapolis, USA). All cells were kept at 37°C and 5% CO_2_, and routinely screened for the absence of mycoplasma.

### Confocal immunofluorescence microscopy

Cells were fixed and permeabilized as described recently [25]. Primary antibodies were used at following dilutions: anti-Smad2/3 (Transduction Laboratories, Lexington, UK), 1:100; anti-desmin (DAKO Corp., Carpinteria, CA, USA), 1:100. After application of cye-dye conjugated secondary antibodies (Jackson Laboratories, West-Grove, USA), imaging of cells was performed with a TCS-SP confocal microscope (Leica, Heidelberg, Germany). Nuclei were visualized using To-PRO3 at a dilution of 1:10,000 (Invitrogen, Carlsbad, USA).

### Measurement of intracellular ROS

Intracellular ROS was measured as previously described [43] with minor modifications. Briefly, cells were plated in 12 well plates and treated with TGF-β1 for the indicated time. For measurement, cells were incubated for 1 hour with 2.5 μM of the oxidation-sensitive probe 2'7'-dichlorodihydrofluorescein diacetate (DCFH-DH) (Invitrogen, Carlsbad, USA) in DMEM plus 10% FCS. Cellular fluorescence intensity was measured at 485/20 and 530/25 nm with Fluorimeter (Wallace) and depicted in percentage with respect to control, as represented by untreated M1-4HSCs.

Diphenyleneiodonium chloride (DPI; Sigma) was used at a final concentration of 20 μM. M1-4HSCs have been treated with TGF-β1 for 3 hours or co-incubated with DPI (4 hours) and TGF-β (3 hours) during overall starvation of 6 hours. Cellular fluorescence intensity was again depicted in percentage with respect to control, represented by 6 hours starved M1-4HSCs.

### Analysis of NADPH oxidase activity

Cells were harvested by trypsinization, pelleted by centrifugation at 2,500 g for 5 min at 4°C, and resuspended in PBS, followed by incubation with 250 μmol/l NADPH. NAD(P)H oxidase activity was analyzed as previously described [31]. NADPH consumption was monitored by the decrease in absorbance at λ = 340 nm for 5 min. For analysis of specific NADPH oxidase activity, the rate of consumption of NADPH inhibited by DPI was measured by adding 10 μmol/l DPI 30 min prior to measurement. For normalization, protein concentration was determined by lysis of an aliquot of cells by adding SDS and protein measurement by Lowry solution. The absorption extinction coefficient used to calculate the amount of NADPH consumed was 6.22 mM^-1 ^cm^-1^. Results were expressed as pmol/l of substrate per minute per milligram of protein.

### Glutathione determination

Cells were washed twice, scraped in PBS at 90% density and centrifuged at 950 g for 5 min at 4°C. Cellular glutathione was extracted in a buffer containing 0.2% Triton X-100, 2.5% sulfosalicylic acid, and then centrifuged at 10,000 g for 10 min at 4°C. The supernatant was used for determination of total (GSH and GSSG) glutathione by the Griffith's method, modified as described previously [44,45]. Using glutathione as standard, glutathione content is expressed as pmol/μg protein and represented as percentage with respect to untreated M1-4HSCs (control).

### Analysis of superoxide dismutase activity

Enzyme activity was determined as previously described [31]. Briefly, cells were harvested as described for glutathione determination. Pellets were lysed in 150 μl 50 mM di-sodiumphosphate buffer containing 0.5% Triton X-100, 1 mM PMSF and 5 μg/ml Leupeptin and sonicated. Lysates were purified by centrifugation at 13,000 g for 10 min at 4°C. SOD activity was measured by monitoring the autooxidation of 6-hydroxy-dopamine. Autooxidation is inhibited by 6-hydroxy-dopamine consuming superoxide generated during this process, as described previously [31,46]. Briefly, the kinetics of autooxidation of 6-hydroxy-dopamine were monitored by λ = 490 nm for 60 sec under conditions that resulted in linear kinetics. Assays of protein extracts (20–30 μg protein in 20 μl protein extract) were carried out under conditions that resulted in 40% – 60% inhibition of the autooxidation of 6-hydroxy-dopamine. Measurements were repeated three times. Data were calculated as percentage of inhibition of the autooxidation of 6-hydroxy-dopamine that was obtained with 10 μg protein. The values are depicted as percentage with respect to untreated M1-4HSCs (control).

### Analysis of catalase activity

Cell harvest and protein extract preparation was performed as described for SOD activity measurement. Catalase activity was measured by monitoring the disappearance of hydrogen peroxide at λ = 240 nm [46]. The reaction mixture contained 40 – 80 μg protein, 50 mmol/l potassium phosphate buffer, pH 7.0, and 10 mmol/l H_2_O_2_. Changes in absorbance were measured for 100 sec. The specific activity was calculated as previously described [31] and depicted as percentage with respect to untreated M1-4HSCs (control).

### Reverse transcription polymerase chain reaction (RT-PCR)

The extraction of poly(A)+ mRNA, reverse transcription to cDNA and PCR were performed as described previously [47]. The conditions for the linear PCR reaction were optimized for each primer pair. The oligonucleotide forward and reverse primers correspond to mouse catalase (5'-CAA CGC TGA GAA GCC TAA-3' and 5'-CGC ACA GCA CAG GAA TAA-3'), c-fos (5'-GCT GAC AGA TAC ACT CCA AGC GG-3'and 5'-AGG AAG ACG TGT AAG TAG TGC AG-3'), γ-glutamylcysteine synthetase (5'-CCT CAT TCC GCT GTC CAA-3' and 5'-CTG CAC ACG CCA TCC TAA-3'), GSPH-1 (5'-TTC GGA CAC CAG GAG AAT-3' and 5'-GCA GCC AGT AAT CAC CAA-3'), GSSG reductase (5'-GCG TGG AGG TGT TGA AGT and 5'-TTC ACC GCT ACA GCG AAG-3'), c-jun (5'-AGA GTT GCA CTC ACT GTG GCT GAA-3' and 5'-AGA ACA GTC CGT CAC TTC AC-3'), Nox4 (5'-TTGCTACTGCCTCCATCAAG-3' and Nox4 5' ATCAACAGCGTGCGTCTAAC-3'), p47^phox ^(5'-CCG AGG CTC ACA TCT GTA-3' and 5'-CAC CAG CTC GTG TCA AGT-3'), PDGF-Rα (5'-CAG ACT TCG GAA GAG AGT GCC ATC-3' and 5'-CAG TAC AAG TTG GCG CGT GTG G-3'), PDGF-Rβ (5'-CCT GAA CGT GGT CAA CCT GCT-3' and 5'-GGC ATT GTA GAA CTG GTC GT-3'), RhoA (5'-GTG GAA TTC GCC TTG CAT CTG AGA AGT-3' and 5'-CAC GAA TTC AAT TAA CCG CAT GAG GCT-3'), SOD 1 (5'-AGC GGT GAA CCA GTT GTG-3' and 5'-CGG CCA ATG ATG GAA TGC-3') and SOD 2 (5'-ACA ACT CAG GTC GCT CTT-3' and 5'-AGC AGG CAG CAA TCT GTA-3'). The specific amplicons were analyzed by agarose gel electrophoresis and visualized with ethidium bromide.

### Statistics

All results are expressed as mean ± standard error of the median (S.E.M.). Comparisons to control, as represented by untreated M1-4HSCs, were performed using Student's *t*-test in case of Figure 2B. With regard to all other data, statistical analyses were performed using ANOVA followed by the post-hoc Duncan test. All data showed normal distribution as analyzed by the Kolmogorov-Smirnov test.

## Competing interests

The author(s) declare that they have no competing interests.

## Authors' contributions

VP performed most of the experiments and also drafted the manuscript. ICC and MMM carried out measurements on NADPH oxidase activity and supported VP by preparing cellular extracts and statistical analyses. HH performed immunofluorescence analyses. IF participated in the design of the study and was involved with the particular expertise on oxidative stress. WM coordinated the study and finally edited the manuscript. All authors have read and approved the content of the manuscript.
